# Potential application of TurboID-based proximity labeling in studying the protein interaction network in plant response to abiotic stress

**DOI:** 10.3389/fpls.2022.974598

**Published:** 2022-08-16

**Authors:** Kaixin Zhang, Yinyin Li, Tengbo Huang, Ziwei Li

**Affiliations:** ^1^Guangdong Provincial Key Laboratory for Plant Epigenetics, College of Life Sciences and Oceanography, Shenzhen University, Shenzhen, China; ^2^Key Laboratory of Optoelectronic Devices and Systems of Ministry of Education and Guangdong, College of Physics and Optoelectronic Engineering, Shenzhen University, Shenzhen, China

**Keywords:** plant, abiotic stress, protein interaction, TurboID, regulation network

## Abstract

Abiotic stresses are major environmental conditions that reduce plant growth, productivity and quality. Protein-protein interaction (PPI) approaches can be used to screen stress-responsive proteins and reveal the mechanisms of protein response to various abiotic stresses. Biotin-based proximity labeling (PL) is a recently developed technique to label proximal proteins of a target protein. TurboID, a biotin ligase produced by directed evolution, has the advantages of non-toxicity, time-saving and high catalytic efficiency compared to other classic protein-labeling enzymes. TurboID-based PL has been successfully applied in animal, microorganism and plant systems, particularly to screen transient or weak protein interactions, and detect spatially or temporally restricted local proteomes in living cells. This review concludes classic PPI approaches in plant response to abiotic stresses and their limitations for identifying complex network of regulatory proteins of plant abiotic stresses, and introduces the working mechanism of TurboID-based PL, as well as its feasibility and advantages in plant abiotic stress research. We hope the information summarized in this article can serve as technical references for further understanding the regulation of plant adaptation to abiotic stress at the protein level.

## Introduction

Plant life needs certain natural factors such as temperature, moisture and nutrition, while plants often suffer from environmental stresses during development including changes of temperature, salinity, water, light, nutrient availability, and toxic chemicals (Vanstraelen and Benkova, 2012; Skalak et al., 2021; Zhou et al., 2021). In response to these abiotic stresses, various adaptations have evolved in plants at the physiological, molecular, and cellular levels, which are regulated by complex signal transduction pathways (Zhu, 2016). In these important pathways, regulatory proteins play an essential role. Proteins rarely act on their own, while often function as complexes through protein-protein interactions (PPIs) (Struk et al., 2019). Therefore, the study of PPIs can not only infer the protein functions within the cell, but also uncover unidentified proteins from their interactions with known proteins (Zhang et al., 2010). There are two types of PPIs, namely constitutive and regulative, in the cell (Fujikawa et al., 2014). Constitutive PPIs are typically ubiquitous and strong interactions, whereas regulative PPIs occur only in certain cellular or developmental contexts or in response to specific incentives (Morsy et al., 2008). The dynamic changes of regulative PPIs confer cells with the ability to rapidly respond to intracellular and extracellular stimuli (Syafrizayanti et al., 2014). Regulative PPIs have the features of instantaneity, specificity and instability, which make them challenging to be studied (Lalonde et al., 2008).

Many classic PPI approaches, such as Yeast Two-Hybrid (Y2H), Co-Immunoprecipitation (Co-IP), Affinity Purification (AP), Pull-down, Bimolecular Fluorescence Complementation (BiFC), and Split Luciferase (Split-LUC), have been utilized for studying the protein interaction network in plant response to abiotic stresses. These techniques have identified many critical regulatory proteins involved in abiotic stress responses (Urano et al., 2010; Li et al., 2016; Zhang et al., 2016; Han et al., 2020; Qin L. et al., 2021). However, they also have many limitations, which hinder their applications, particularly in the analyses of regulative PPIs. Recently, a new PPI technique named TurboID-based proximity labeling (PL) has been applied in bio-research, which has a number of advantages especially for studying dynamic and transient PPIs (Bohnert et al., 2006; Branon et al., 2018; Zhang et al., 2019; Cho et al., 2020; Li et al., 2021). Although there are only a few cases of its application in plant research, we envision wide usage of this cutting-edge technique in dissecting the protein interaction network that regulates abiotic stress responses in plants.

## Classic protein-protein interaction approaches to study plant response to abiotic stresses

### Principles of classic protein-protein interaction approaches and their applications in studying plant abiotic stress responses

Classic PPI approaches, including Y2H, Co-IP, AP, Pull-down and BiFC, have been widely utilized in plant studies and introduced in detail in a number of review articles (Drewes and Bouwmeester, 2003; Weinthal and Tzfira, 2009; Zhang et al., 2010; Braun et al., 2013; Ferro and Trabalzini, 2013; Rao et al., 2014). The Y2H system includes bait and prey proteins in frame with DNA-binding domain (BD) or a transactivation domain (AD), respectively. When AD and BD domains are in spatial proximity to each other, expression of the reporter gene is activated to demonstrate the interaction between bait and prey (Causier and Davies, 2002). Co-IP and AP are two *in vivo* PPI approaches under near-physiological conditions. Co-IP and AP have similar principles, which entail overexpression of the bait protein (with or without an affinity tag) in plant protoplast or tissue, and isolation of the bait with its interacting partners (prey) through purification based on antibody-antigen interactions. The isolated bait-prey complex can be analyzed by liquid chromatography tandem-MS (LC-MS/MS) to achieve high throughput analysis (Ransone, 1995; Masters, 2004; Xie et al., 2012; Xing et al., 2016). Pull-down is an *in vitro* method that can be used to detect or validate the direct interaction between bait and prey proteins. In Pull-down approach, bait or prey protein is usually expressed as a fusion protein with tags in bacteria, the immobilizing bait-tag fusion protein on tag specific column is used as affinity support to catch and purify the prey proteins that interact with bait protein, and these prey proteins can be detected by sodium-dodecyl sulfate polyacrylamide gel electrophoresis (SDS-PAGE) and analyzed by western-blotting detection (Louche et al., 2017). BiFC and Split-LUC are based on the principle of fluorescent protein-fragment complementation assay (PCA). The individual N- or C- terminal part of a fluorescent protein normally has no fluorescence signal, but when N- and C- terminal parts are fused with two partner proteins, respectively, interaction of these two proteins will make the N- and C- parts close enough to regain the fluorescent protein structure and activity (Kerppola, 2006, 2008; Azad et al., 2014). The results of BiFC and Split-LUC can both be shown by the emission of the reconstructed fluorescent proteins, and Split-LUC signals can also be quantified by the luciferase activity assay (Kerppola, 2006, 2008; Azad et al., 2014).

Abiotic stresses such as temperature extremes, salinity, drought, reduced nutrient availability, and toxic chemicals are major limiting factors for plant development. Plants have evolved excellent defense mechanisms to protect themselves from abiotic stresses including stress sensing, signal transduction and transcriptional regulation, etc., (Zhang H. et al., 2022). The classic PPI approaches mentioned above are widely used to build up the protein interaction network and identify hub proteins, including transcription factors, signaling molecules and transporter proteins in the regulation of plant response to abiotic stresses (Supplementary Table 1). For example, in phosphorus (Pi) deficiency stress, the interaction of the Ubiquitin-Conjugating Enzyme PHO2 and the Pi transporter PHTs had been verified by Y2H and BiFC (Liu et al., 2012), and the feedback inhibition of SPX-domain proteins on PHOSPHATE STARVATION RESPONSE 1 (PHR1), the central transcriptional regulator of Pi Starvation Responses (PSR), had also been assessed by Y2H, Co-IP and BiFC (Lv et al., 2014). These results greatly contributed to the understanding of the genetic network that controls PSR in plants.

### The advantages and disadvantages of the classic protein-protein interaction approaches

Although the classic PPI approaches have been successfully applied in many abiotic stress studies, their shortages cannot be overlooked. For example, Y2H has benefits of high sensitivity, maintaining the natural folding of fusion proteins and convenient operation that bypasses the complicated steps of protein extraction and purification. However, it also has obvious disadvantages, including high technical false positive rates due to the strong spontaneous activation of reporter gene transcription, and toxicity of plant proteins to yeast cells, which can cause false negative results. Also, Y2H often fails to detect protein interactions that rely on post-translational modifications (PTMs). Therefore, Y2H is more suitable for cDNA library screening rather than confirming protein interactions, and the Y2H results often need to be confirmed by other PPI approaches (Hamdi and Colas, 2012; Mehla et al., 2015). Unlike Y2H, Co-IP/AP-MS can be used to pull down protein complexes under native physiological conditions to reflect the *in vivo* binding. But these approaches also suffer from high false positive rates. Also, Co-IP/AP-MS need to overexpress the bait protein which may influence its physiological properties. In addition, the choice of lysis conditions may have strong influence on the result of Co-IP/AP-MS. Lysis conditions may break PPI, and the low solubility of some subcellular structures in normal lysis buffer, e.g., plasmalemma, cytoskeleton and nucleus, may lead to negative results as well. Another shortcoming of Co-IP/AP-MS is that the instantaneous interactions or weak interactions often fail to be detected, and it is unable to distinguish direct and indirect interactions between the examined proteins. Pull-down is an approach used to detect the direct interaction between two proteins *in vitro*, with the outstanding features of being quick, sensitive, and quantifiable. But there are some disadvantages of Pull-down, such as it cannot reflect the protein interactions in plant physiological conditions, and each experiment needs to be optimized to keep characterized interactions from artifacts (Struk et al., 2019). Comparing to Co-IP/AP-MS or Pull-down, BiFC and Split-LUC have the advantage in identifying weak and instantaneous interactions because of the stability of the reconstituted GFP/YFP or LUC complexes. BiFC can also reveal the cellular localization of the PPI complex, which is convenient for further cellular studies. However, BiFC and Split-LUC can only be used to investigate the interaction of two proteins, and the interaction might be influenced by protein conformation, which could be changed after the joining of the N- and C- terminus of fluorescent proteins. These factors all limit the application of BiFC and Split-LUC in high throughput PPI analyses (Kerppola, 2008).

## Application of TurboID-based proximity labeling in studying plant abiotic stress responses

### Mechanisms and advantages of TurboID-based proximity labeling technique

Due to the above disadvantages of classic PPI approaches and to avoid spurious results from heterologous expression, instantaneous and weak interactions, and indistinguishable cellular localization of the target proteins, enzyme-catalyzed PL techniques have been developed as novel alternative approaches to study PPIs (Qin W. et al., 2021; Xu et al., 2021; Yang et al., 2021; Mair and Bergmann, 2022). TurboID, a biotin ligase, has been exploited as an important PL enzyme with the advantages of non-toxicity and high catalytic efficiency (May et al., 2020).

TurboID is a 35 kDa biotin ligase engineered by yeast display-based directed evolution, which has 15 mutations relative to the wild-type *Escherichia coli* biotin ligase (BirA) (Branon et al., 2018). By fusing TurboID with the target protein of interest and expressing it in cells, when biotin is supplied in the presence of ATP, TurboID catalyzes biotin and forms reactive biotinoyl-5′-AMP (bioAMP) from biotin and ATP. These free bioAMPs are released and diffused to the vicinity of the target protein, which can covalently bind to lysine residues of proteins that are in close proximity to the TurboID enzyme (Roux et al., 2012; Branon et al., 2018). The biotin-labeled proteins are enriched and affinity purified by streptavidin pulldown and subsequently identified by MS, so as to identify the proximal proteins of the target protein (Roux et al., 2012). In contrast to classic methods, TurboID-based PL adds covalently bound tag in living cells, such that spatial relationships and interaction networks are not disrupted. In addition, the TurboID-based PL system simply requires a supply of exogenous non-toxic biotin, which permits it to be applied *in vivo* without causing damage to living cells. Furthermore, TurboID has high catalytic efficiency and biotinylation of proximal proteins can be completed in living cells within 10 min at 25°C, which allows its quick application in plants grown under ambient conditions (Branon et al., 2018; Mair et al., 2019; May et al., 2020; Zhang et al., 2020). Most importantly, TurboID can identify weak and transient protein interactions in living cells, which frequently fail to be captured by classic Co-IP/AP approaches (Branon et al., 2018; Kim et al., 2019). Moreover, it can also identify rare protein complexes or local organelle proteomes in individual cell types of complex multicellular organisms (Branon et al., 2018; Mair et al., 2019).

### Application of TurboID-based proximity labeling in plant research

TurboID-based PL techniques have been applied successfully in a number of biological studies. For example, TurboID-based PL has been used to map local proteomes and screen novel interactors *in vivo* in zebrafish (Xiong et al., 2021). TurboID biotin ligase can also efficiently tag the entire proteome of specific cell types in the mouse brain, and dynamically track and identify tissue-specific or stimulation-specific secretory proteins in living body (Chua et al., 2021; Kim et al., 2021; Sun et al., 2022). TurboID-based PL was also used to identify host proteins interacting with viruses including coronavirus and syndrome coronavirus-2 (SARS-CoV-2), which help to elucidate the mechanism of virus infection and provide resources for the development of antiviral drugs for coronavirus disease 2019 (COVID-19) treatment (V’Kovski et al., 2020; Zhang Y. et al., 2022).

In plant research, TurboID-based PL technology has also been utilized in a variety of systems to study PPIs. For example, TurboID-based PL was used to identify interactors of a plant immune receptor N, which is a Nucleotide-binding Leucine-rich Repeat (NLR) that confers plant resistance to Tobacco Mosaic Virus (TMV) in *Nicotiana benthamiana*. In this work, a new regulator Ubiquitin Protein Ligase E3 Component N-Recognin 7 (UBR7) was found, which directly interacts with N and mediates immunity against plant pathogens (Zhang et al., 2019). TurboID-based PL was also applied to identify partners of the stomatal-specific transcription factor FAMA and help to obtain the nuclear proteome of young guard cells in *Arabidopsis thaliana* seedlings, which demonstrate that TurboID-based PL can be used to detect interactions of low abundant proteins and local proteomes of rare plant cell types (Mair et al., 2019). In addition, TurboID-based PL was also used to characterize neighboring proteins of Brassinosteroid-Insensitive 2 (BIN2), the regulatory kinase of Brassinosteroid (BR) pathways. This study uncovered a suite of previously unidentified BIN2 proximal proteins, which further enriched BIN2-mediated BR signaling networks (Kim et al., 2019). Furthermore, TurboID-based PL was successfully used to identify multiple interacting proteomes in the cell suspension cultures of tomato (*Solanum lycopersicum*), *N. benthamiana* and *Arabidopsis*, which showed that this technology can effectively capture membrane-associated protein interactions in different plant model systems (Arora et al., 2020).

## Technical feasibility and advantages of TurboID in the studies of abiotic stresses

Abiotic stresses can be sensed by plants not only at the cell surface, such as by receptors at the cell wall and plasma membrane, but also in intracellular compartments, such as by signaling proteins in the cytoplasm and nucleus. Stress signaling triggers physical or chemical changes of biomolecules in the plant cell, which can lead to a cellular stress response (Zhang H. et al., 2022). Signal transduction in this process involves secondary messengers and regulatory proteins, and the interactions between the components of signaling pathways tend to be transient and dynamic. For example, many kinases in the Mitogen-Activated Protein Kinase (MAPK) signal transduction cascades can be rapidly activated by abiotic stresses. These kinases can affect their own activities by interacting with specific partner proteins and can also modulate the activities of substrates through transient kinase-substrate interactions, thus dynamically acting in various physiological processes and regulating plant tolerance to abiotic stresses (Mishra et al., 2006; Moustafa et al., 2014; Andrasi et al., 2019). In addition, the stress-related PTMs, including phosphorylation, glycosylation, ubiquitination, sumoylation, oxidation, carbonylation and nitrosylation, etc., are also modulated by transient enzyme-substrate interactions because of the rapid turnover of the corresponding enzymes (Wu et al., 2016). Understanding these dynamic and transient PPIs are one of the major challenges in the investigation of the stress signaling network in plants. In this sense, the high efficiency of TurboID-based PL in detecting dynamic and transient protein interactions will make it a particularly useful tool in studying abiotic stress responses in plants. Furthermore, as many regulatory proteins acting in stress signal transduction pathways, such as transcription factors, transmembrane receptors and kinases are in low abundance (Kosova et al., 2011; Abreu et al., 2013), the advantage of TurboID-based PL in capturing low-abundant proteins will also greatly contribute to the identification of stress related factors at the protein level.

Abiotic stress causes multilevel responses, including stress sensing, signal transduction, transcription, transcript processing, translation and PTMs (Zhang H. et al., 2022). These responses can be initiated in various cellular structures including plasma membrane, nucleus, mitochondria, chloroplast, endoplasmic reticulum (ER) and cell wall. The important functions of these cellular structures in stress responses and the involvement of protein interactions in the regulation of their activities suggest that organelle proteome analysis may provide key information of the cellular mechanisms of plant response to stresses (Couee et al., 2006; Nouri and Komatsu, 2010; Pang et al., 2010; Hüner et al., 2012; Komatsu et al., 2012; Yin and Komatsu, 2016). However, due to the dynamic state of organelles and their proteins, clarifying the subcellular distribution and expression of organelle proteins has always been a challenging task (Boisvert et al., 2010). Utilization of TurboID-based PL may open a new avenue for organ and subcellular proteome research. TurboID-based PL has been used to label proteins located in the cell membrane, mitochondrial matrix, cytoplasm, nucleus, and ER lumen/membrane in mammalian cells (Branon et al., 2018; May et al., 2020). It can also be used to efficiently and specifically detect the proteome of different subcellular components of plants, such as the nuclear proteome of stomatal guard cells in *Arabidopsis* (Mair et al., 2019). Compared with classic tools, TurboID-based PL technology can label the organelle proteome of interest in living cells without isolating tissues and organelles, therefore, it can be used to investigate non-membrane-enclosed organelles that cannot be purified by classic biochemical fractionation methods. Another important application of the TurboID-based PL technology is that it can be used in combination with fluorescent microscopy to assess the cellular compartmentalization information of protein interactions. Because one protein can display diverse functions depending on its subcellular localization (Kosova et al., 2018), this application is helpful for understanding the spatial-specific regulatory process in cells in response to abiotic stress.

## Conclusion and perspective

**FIGURE 1:**
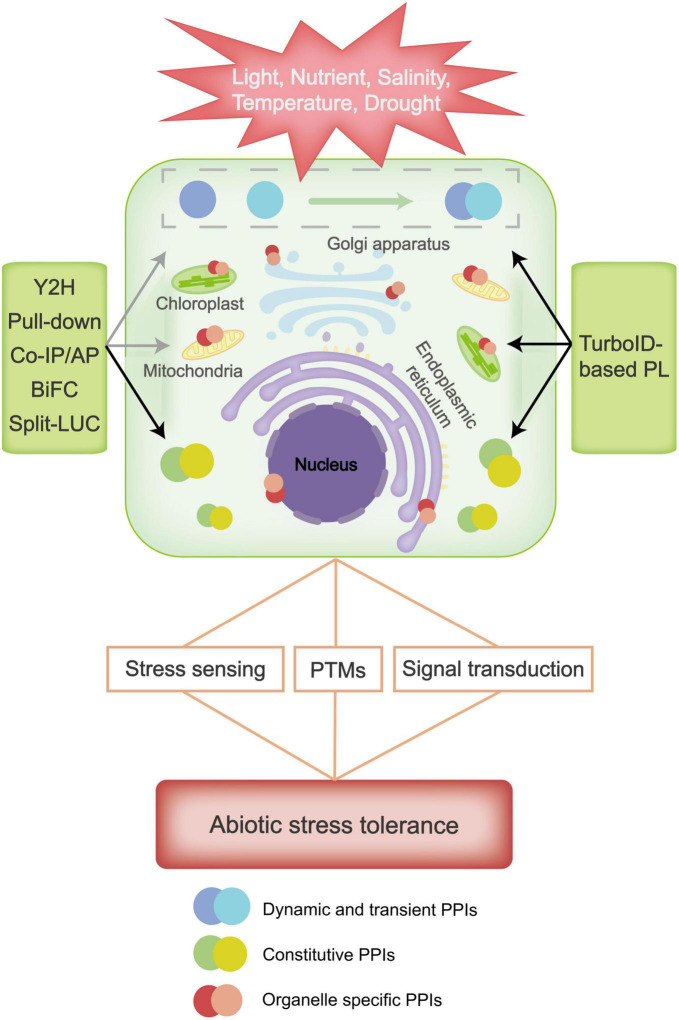
Plant resistance to abiotic stresses involves stress sensing, signal transduction and post-translational modifications (PTMs) of proteins, etc., in which many protein-protein interactions (PPIs) are involved. Classic methods such as Yeast Two Hybrid (Y2H), Pull-down, Co-Immunoprecipitation (Co-IP), Affinity Purification (AP), Bimolecular Fluorescence Complementation (BiFC), and Split Luciferase (Split-LUC) can easily detect stable protein interactions, such as constitutive PPIs that are typically macromolecular complex, but they are difficult to detect dynamic and organelle specific interacting proteins in response to abiotic stress. TurboID-based proximity labeling (PL) has great advantages in detecting dynamic, transient and organelle specific interacting proteins, and can be applied to study regulative PPIs under abiotic stress. Black and gray arrows indicate high and low applicability of the classic and TurboID-based technique(s) in detecting the corresponding PPIs.

Plants have evolved excellent defense mechanisms to protect themselves from abiotic stresses. Classic PPI approaches like Y2H, Co-IP/AP-MS, Pull-down, BiFC and Split-LUC have contributed to identification of stress-regulatory proteins in plants (Figure 1). However, due to their limitations in detecting weak instantaneous interactions, distinguishing cellular localizations and directly assessing protein interactions in subcellular organs, further application of classic PPIs in systematic studies of plant stress responses is largely hindered. As a recently developed PPI approach, TurboID-based PL has been applied in mapping PPIs in a variety of species and has proven especially useful in dissecting signaling pathways (Kim et al., 2019). TurboID-based PL has a number of advantages, such as high flexibility, easy implementation, and great efficiency in detecting protein interactors that are low abundant, transient and specifically expressed in organelles. All of these advantages may greatly help us in our study of the mechanisms of plant stress responses (Kim et al., 2019; Mair et al., 2019; Figure 1). However, TurboID-based PL also has its own limitations, which results in their non-applicability in some abiotic stress conditions. For instance, biotin ligase activity is markedly influenced by low temperature, so TurboID-based PL is not suitable for studying cold stress responses. Also, the proximity-dependent labeling method only provide information on which proteins are in proximity to each other, it does not show direct evidence for a physical interaction between these proteins. Therefore, TurboID-based PL may needs to be combined with classic PPI approaches to map protein interactions in plant responses, and new proximity labeling ligases should be developed to overcome these shortcomings of TurboID. Overall, with the continuous renovation of PPI approaches, we believe the future research on protein interactions will provide in-depth knowledge of systematic molecular mechanisms for plant abiotic stress responses.

## Author contributions

KZ, YL, TH, and ZL were contributed to the writing of this review. All authors contributed to the article and approved the submitted version.
