# Supplementary material for: Potential application of TurboID-based proximity labeling in studying the protein interaction network in plant response to abiotic stress
Source: Front Plant Sci. 2022 Aug 16;13:974598. doi: 10.3389/fpls.2022.974598 (PMC9426856; doi:10.3389/fpls.2022.974598)
Supplement: Supplementary file 1 [file Table_1.docx]

*Supplementary Material*

Application of TurboID-based proximity labeling in studying the protein interaction network in plant response to abiotic stress

Kaixin Zhang^1,2^, Yinyin Li^1^, Tengbo Huang^1^, Ziwei Li^1*^

^1^ Guangdong Provincial Key Laboratory for Plant Epigenetics, College of Life Sciences and Oceanography, Shenzhen University, Shenzhen 518055, China

^2^ Key Laboratory of Optoelectronic Devices and Systems of Ministry of Education and Guangdong,

College of Physics and Optoelectronic Engineering, Shenzhen University, Shenzhen 518055, China

*** Correspondence:**Corresponding Author
liziwei1989@126.com (Ziwei Li)

**Supplementary Table**

Table 1 Core regulation proteins in abiotic stress regulation which first identified in model plant through classic PPI approaches.

| **Abiotic stress** | | **Regulation protein module in abiotic stress response** | **Techniques** | **Organism** | **Reference** |
| --- | --- | --- | --- | --- | --- |
| Temperature | Heat | HSP90-HsfA1 | BiFC, Pull-down | *A. thaliana* | (Yamada et al., 2007) |
|  |  | CBK3-HsfA | Y2H | *A. thaliana* | (Liu et al., 2008) |
|  |  | CaM-PP7-HSF | Y2H | *A. thaliana* | (Liu et al., 2007) |
|  |  | ELF3-ELF4 | Y2H | *A. thaliana* | (Jung et al., 2020) |
|  |  | MPK6-HsfA2 | IP | *A. thaliana* | (Evrard et al., 2013) |
|  |  | DPB3-DREB2A; NF-YA2-NF-YB3 | Y2H, BiFC, Co-IP, Pull-down | *A. thaliana* | (Sato et al., 2014) |
|  |  | NF-YA2-NF-YB3-DPB3 | Y2H | *A. thaliana* | (Sato et al., 2014) |
|  | Chilling | MEKK1-MKK2-MPK4 | Y2H, IP | *A. thaliana* | (Teige et al., 2004) |
|  |  | CRLK1- MEKK1 | Co-IP, BiFC, Pull-down | *A. thaliana* | (Yang et al., 2010) |
|  |  | MEKK1- MKK2 | IP | *A. thaliana* | (Furuya et al., 2013) |
|  |  | MKK4/5-MPK3/6- ICE1 | IP | *A. thaliana* | (Zhao et al., 2017) |
|  |  | NPR1-HsfA1 | BiFC, Co-IP | *A. thaliana* | (Olate et al., 2018) |
|  | Freeze | SlZ1-ICE1 | IP | *A. thaliana* | (Miura et al., 2007) |
|  |  | OST1/SnRK2.6-ICE1; HOS1-ICE1 | Y2H, Pull-down, Co-IP | *A. thaliana* | (Ding et al., 2015) |
|  |  | MAPKKK-MAKK5-MPK3/6-ICE1 | Y2H, Pull-down, Co-IP | *A. thaliana* | (Li et al., 2017) |
|  |  | CRPK1-14-3-3 proteins | Y2H, Pull-down, Co-IP | *A. thaliana* | (Liu et al., 2017) |
|  |  | 14-3-3 proteins-CBFs | IP-MS, Co-IP, Pull-down | *A. thaliana* | (Liu et al., 2017) |
|  |  | OST1/SnRK2.6-PUB25/26-MYB15 | Y2H, Co-IP, Split-LUC, Pull-down | *A. thaliana* | (Wang et al., 2019) |
|  |  | NMT1-ERG2-OST1 | Y2H, Co-IP, Split-LUC, Pull-down | *A. thaliana* | (Ding et al., 2019) |
|  |  | OST1-BTF3L-CBF | Y2H, Co-IP, Split-LUC, BiFC, Pull-down | *A. thaliana* | (Ding et al., 2018) |
|  |  | BIN2-ICE1 | Y2H, Co-IP, BiFC, IP, Pull-down | *A. thaliana* | (Ye et al., 2019) |
| Salinity | Na^+^ ionic | LRX3/4/5-RALF22/23-FER | IP-MS, Co-IP, Split-LUC, Pull-down | *A. thaliana* | (Zhao et al., 2018) |
|  |  | ANN4-SOS2-SCaBP8 | Y2H, BiFC, Co-IP, Split-LUC | *A. thaliana* | (Ma et al., 2019) |
| Light | UV-B | COP1-SPA | Co-IP | *A. thaliana* | (Huang et al., 2013) |
|  |  | UVR8-COP1-SPA | Y3H, Co-IP | *A. thaliana* | (Huang et al., 2013) |
|  |  | RUP1/RUP2-UVR8 | Co-IP | *A. thaliana* | (Heijde and Ulm, 2013) |
|  |  | UVR8-WRKY36 | Y2H, Co-IP, BiFC, Pull-down | *A. thaliana* | (Yang et al., 2018) |
|  |  | PIF-COP1 | Co-IP | *A. thaliana* | (Sharma et al., 2019) |
| Nutrient | N | NAR2.1-NRT2.1/2.2/2.3a | Y2H | *O. sativa* | (Yan et al., 2011) |
|  |  | CIPK23-AMT1;1/ AMT1;1 | Y2H, BiFC | *A. thaliana* | (Straub et al., 2017) |
|  |  | CIPK23-NRT1.1 | BiFC | *A. thaliana* | (Leran et al., 2015) |
|  | P | SPX1-PHR1 | Y2H, Co-IP | *A. thaliana* | (Puga et al., 2014) |
|  |  | SPX4-PHR2 | Y2H, Co-IP, BiFC, Pull-down | *O. sativa* | (Lv et al., 2014) |
|  |  | SPX6-PHR2 | Y2H, Co-IP, BiFC | *O. sativa* | (Zhong et al., 2018) |
|  |  | PHO2-PHO1/PHT1 | Y2H, BiFC | *A. thaliana* | (Liu et al., 2012) |
|  |  | SDEL1-SPX4 | Y2H, BiFC, Co-IP | *O. sativa* | (Ruan et al., 2019) |
|  |  | NLA1-PT2/PT8 | BiFC, | *O. sativa* | (Yue et al., 2017) |
|  |  | WRKY108-WRKY21 | Y2H, BiFC, Pull-down | *O. sativa* | (Zhang et al., 2021) |
|  |  | ALIX-ESCRT-III | Y2H, BiFC | *A. thaliana* | (Cardona-López et al., 2015) |
|  |  | PRU1-WRKY6 | Y2H, Co-IP, BiFC, Pull-down | *A. thaliana* | (Ye et al., 2018) |
|  |  | CK2α3/β3-PTs | Y2H, Co-IP, Pull-down | *O. sativa* | (Chen et al., 2015) |
|  | K | AKT1-CIPK23 | Y2H | *A. thaliana* | (Kim et al., 2000) |
|  |  | KC1-SYP121 | Y2H, Co-IP, BiFC | *A. thaliana* | (Honsbein et al., 2009) |
|  |  | CBL1/CBL9-CIPK23 | Y2H, BiFC | *A. thaliana* | (Xu et al., 2006) |
|  |  | ILK1-CML9 | Split-LUC, BiFC | *A. thaliana* | (Brauer et al., 2016) |
|  |  | HAK5-ILK1 | Split-LUC, BiFC | *A. thaliana* | (Brauer et al., 2016) |
|  |  | AKT1-KC1 | BiFC | *A. thaliana* | (Wang et al., 2016) |
|  |  | PP2Cs-AKT1 | Y2H | *A. thaliana* | (Lan et al., 2011) |
|  | Ca | CIPK-CBL | Y2H | *A. thaliana* | (Shi et al., 1999) |
|  |  | PP2C-CIPK | Y2H | *A. thaliana* | (Lan et al., 2011) |
|  |  | PP2C-SnRK2 | Co-IP | *A. thaliana* | (Belda-Palazon et al., 2020) |
|  |  | PP2C-RCAR | Y2H | *A. thaliana* | (Tischer et al., 2017) |
|  |  | SnRK2- M3Kδ6 | BiFC, Co-IP | *A. thaliana* | (Takahashi et al., 2020) |
|  |  | SLAH3-CDPK | BiFC | *A. thaliana* | (Geiger et al., 2011) |
|  | Fe | IDEF1-IBP1.1/IBP1.2 | Y2H | *O. sativa* | (Kobayashi et al., 2014) |
|  | Zn | FIT-bHLH38/bHLH39 | Y2H, BiFC | *A. thaliana* | (Yuan et al., 2008) |
|  |  | MTP12-MTP5 | BiFC | *A. thaliana* | (Fujiwara et al., 2015) |
|  | B | BOR1-AP2 | Y2H, Co-IP | *A. thaliana* | (Yoshinari et al., 2019) |
| Toxic | As | PHIF1-PHR1 | Y2H, Co-IP, Pull-down | *A. thaliana* | (Navarro et al., 2021) |
|  | Cd | CUL4-PRL1-MYB43-HMAs | Y2H, BiFC, Co-IP, Pull-down | *A. thaliana* | (Zheng et al., 2022) |
|  |  | ABI5-MYB49 | Y2H, BiFC, Co-IP, Pull-down | *A. thaliana* | (Zhang et al., 2019) |

HSP70/90, Heat Shock Protein 70/90; Hsf, Heat Shock Transcription Factor; CBK3, CaM-Binding Protein Kinases 3; CaM; Calmodulin; ELF3/4; Early Flowering 3/4; MPK6; Mitogen-Activated Protein Kinases 6; DREB2A; Dehydration-Responsive Element Binding Protein 2a; DPB3 DNA Polymerase II Subunit B3; NF-YA2/YB3, Nuclear Factor Y -Subunit A2/B3; PP7; Serine/Threonine Phosphatase 7; MEKK1, MAPK/ERK Kinase Kinase; MKK2/4/5, MAP Kinase Kinase; MPK3/4/6, Mitogen-Activated Protein Kinase; CRLK1, Calcium/Calmodulin-Regulated Receptor-Like Kinase; ICE1, Inducer of CBF Expression 1;,NPR1, Nonexpressor of Pathogenesis-Related genes 1; SlZ1, SAP and Miz; BTF3, Basic Transcription Factor 3; OST1, Open Stomata 1; SnRK2.6, Sucrose Non fermenting 1-Related Protein Kinase 2-6; HOS1, High Expression Of Osmotically Responsive Gene 1;MAPKKK, Mitogen-Activated Protein Kinase Kinase Kinase;CRPK1, Cytoplasmic Receptor-Like Kinase 1; CBFs, C-Repeat Binding Factors; PUB25/26. Plant U Box E3 Ubiquitin; NMT1, N-Myristoyl Transferase; ERG2, Clade-E Growth-Regulating 2; BTF3L, BTF3-Like Protein; BIN2, Brassinosteroid-Insensitive 2; LRX3/4/5, Leucine-Rich Repeat Extension 3/4/5; RALF22/23, Rapid Alkalinization Factor 22/23; FER, Plasma Membrane-Localized Receptor-Like Protein Kinase FERONIA; ANN4, ANNEXIN4; SOS2, Salt Overly Sensitive; SCaBP8, SOS3-Like Calcium-Binding Proteins; COP1, Constitutive Photomorphogenesis 1; SPA, COP1-Suppressor of PHYA; UVR8, UV Resistance Locus 8; RUP1/2, Repressor of UV-B Photomorphogenesis; WRKY, WRKY DNA-Binding Protein; PIF, Phytochrome-Interacting Factor 4/5; NAR2.1, Nitrate Assimilation Related Protein; NRT2.1/2.2/2.3a, Nitrate Transporter 2.1/2.2/2.3a; CIPK23, Calcineurin B-like Calcium Sensor Proteins; AMT1;1, High-Affinity Ammonium Transporters 1;1; SPX1/4/6, SYG1/Pho81/XPR1 domain-containing protein 1/4/6; PHR1/2, Phosphate Starvation Response 1/2; PHO1, Phosphate1; PHT1, Phosphate Transporter 1; PHO2, Ubiquitin-Conjugating Enzyme 24; SDEL1, RING-Finger Domain-Containing E3 ligases; NLA1, Nitrogen Limitation Adaptation 1; PT2/8, Inorganic Phosphate (Pi) Transporters; ALIX, ALG-2 Interacting Protein-X; ESCRT-III, Endosomal Complex Required for Transport; PRU1, Phosphate Response Ubiquitin E3 Ligase1; CK2α3/β3, Casein Kinase II; AKT1, K^+^ Transporter; KC1, K^+^ channel subunit 1; SYP121, Soluble N-ethylmaleimide-Sensitive Factor Protein Attachment Protein Receptor 121; CBL1/9, Calcineurin B-Like 1/9; ILK1, Integrin-Linked Kinase 1; CML9,; Calmodulin-Like Protein 9; HAK5, H^+^/K^+^ Symporter; AKT1, K^+^ Transporter 1; PP2Cs, Protein Phosphatase 2C; RCAR, Regulatory Component of Aba Receptor 1; SnRK2s, Snf1-Related Protein Kinase 2s; M3Kδ6, MAPKK-kinases; CDPK, Calcium-Dependent Protein Kinase 1.

# References

Belda-Palazon, B., Adamo, M., Valerio, C., Ferreira, L.J., Confraria, A., Reis-Barata, D., et al. (2020). A dual function of SnRK2 kinases in the regulation of SnRK1 and plant growth. *Nat. Plants* 6**,** 1345-1353. doi: 10.1038/s41477-020-00778-w

Brauer, E.K., Ahsan, N., Dale, R., Kato, N., Coluccio, A.E., Pineros, M.A., et al. (2016). The raf-like kinase ILK1 and the high affinity K^+^ transporter HAK5 are required for innate immunity and abiotic stress response. *Plant Physiol.* 171**,** 1470-1484. doi: 10.1104/pp.16.00035

Cardona-López, X., Cuyas, L., Marín, E., Rajulu, C., Irigoyen, M.L., Gil, E., et al. (2015). ESCRT-III-associated protein ALIX mediates high-affinity phosphate transporter trafficking to maintain phosphate homeostasis in *Arabidopsis*. *Plant Cell* 27**,** 2560-2581. doi: 10.1105/tpc.15.00393

Chen, J., Wang, Y., Wang, F., Yang, J., Gao, M., Li, C., et al. (2015). The rice CK2 kinase regulates trafficking of phosphate transporters in response to phosphate levels. *Plant Cell* 27**,** 711-723. doi: 10.1105/tpc.114.135335

Ding, Y., Jia, Y., Shi, Y., Zhang, X., Song, C., Gong, Z., et al. (2018). OST1-mediated BTF3L phosphorylation positively regulates CBFs during plant cold responses. *EMBO. J.* 37. doi: 10.15252/embj.201798228

Ding, Y., Li, H., Zhang, X., Xie, Q., Gong, Z., and Yang, S. (2015). OST1 kinase modulates freezing tolerance by enhancing ICE1 stability in *Arabidopsis*. *Dev. Cell* 32**,** 278-289. doi: 10.1016/j.devcel.2014.12.023

Ding, Y., Lv, J., Shi, Y., Gao, J., Hua, J., Song, C., et al. (2019). EGR2 phosphatase regulates OST1 kinase activity and freezing tolerance in *Arabidopsis*. *EMBO. J.* 38. doi: 10.15252/embj.201899819

Evrard, A., Kumar, M., Lecourieux, D., Lucks, J., Koskull-Doring, P.V., and Hirt, H. (2013). Regulation of the heat stress response in *Arabidopsis* by MPK6-targeted phosphorylation of the heat stress factor HsfA2. *PeerJ.* 1**,** e59. doi: 10.7717/peerj.59

Fujiwara, T., Kawachi, M., Sato, Y., Mori, H., Kutsuna, N., Hasezawa, S., et al. (2015). A high molecular mass zinc transporter MTP12 forms a functional heteromeric complex with MTP5 in the Golgi in *Arabidopsis thaliana*. *Febs. J.* 282**,** 1965-1979. doi: 10.1111/febs.13252

Furuya, T., Matsuoka, D., and Nanmori, T. (2013). Phosphorylation of *Arabidopsis thaliana* MEKK1 via Ca(2^+^) signaling as a part of the cold stress response. *J. Plant Res.* 126**,** 833-840. doi: 10.1007/s10265-013-0576-0

Geiger, D., Maierhofer, T., Al-Rasheid, K.A., Scherzer, S., Mumm, P., Liese, A., et al. (2011). Stomatal closure by fast abscisic acid signaling is mediated by the guard cell anion channel SLAH3 and the receptor RCAR1. *Sci. Signal.* 4**,** ra32. doi: 10.1126/scisignal.2001346

Heijde, M., and Ulm, R. (2013). Reversion of the *Arabidopsis* UV-B photoreceptor UVR8 to the homodimeric ground state. *Proc. Natl. Acad. sci. U. S. A.* 110**,** 1113-1118. doi: 10.1073/pnas.1214237110

Honsbein, A., Sokolovski, S., Grefen, C., Campanoni, P., Pratelli, R., Paneque, M., et al. (2009). A tripartite SNARE-K^+^ channel complex mediates in channel-dependent K^+^ nutrition in *Arabidopsis*. *Plant Cell* 21**,** 2859-2877. doi: 10.1105/tpc.109.066118

Huang, X., Ouyang, X., Yang, P., Lau, O.S., Chen, L., Wei, N., et al. (2013). Conversion from CUL4-based COP1-SPA E3 apparatus to UVR8-COP1-SPA complexes underlies a distinct biochemical function of COP1 under UV-B. *Proc. Natl. Acad. Sci. U. S. A.* 110**,** 16669-16674. doi: 10.1073/pnas.1316622110

Jung, J.H., Barbosa, A.D., Hutin, S., Kumita, J.R., Gao, M., Derwort, D., et al. (2020). A prion-like domain in ELF3 functions as a thermosensor in *Arabidopsis*. *Nature* 585**,** 256-260. doi: 10.1038/s41586-020-2644-7

Kim, K.N., Cheong, Y.H., Gupta, R., and Luan, S. (2000). Interaction specificity of *Arabidopsis* calcineurin B-like calcium sensors and their target kinases. *Plant Physiol.* 124**,** 1844-1853. doi: 10.1104/pp.124.4.1844

Kobayashi, T., Itai, R.N., and Nishizawa, N.K. (2014). Iron deficiency responses in rice roots. *Rice* 7**,** 27. doi: 10.1186/s12284-014-0027-0

Lan, W.Z., Lee, S.C., Che, Y.F., Jiang, Y.Q., and Luan, S. (2011). Mechanistic analysis of AKT1 regulation by the CBL-CIPK-PP2CA interactions. *Mol. Plant* 4**,** 527-536. doi: 10.1093/mp/ssr031

Leran, S., Edel, K.H., Pervent, M., Hashimoto, K., Corratge-Faillie, C., Offenborn, J.N., et al. (2015). Nitrate sensing and uptake in *Arabidopsis* are enhanced by ABI2, a phosphatase inactivated by the stress hormone abscisic acid. *Sci. Signal.* 8**,** ra43. doi: 10.1126/scisignal.aaa4829

Li, H., Ding, Y., Shi, Y., Zhang, X., Zhang, S., Gong, Z., et al. (2017). MPK3- and MPK6-mediated ICE1 phosphorylation negatively regulates ICE1 stability and freezing tolerance in *Arabidopsis*. *Dev. Cell* 43**,** 630-642.e634. doi: 10.1016/j.devcel.2017.09.025

Liu, H.T., Gao, F., Li, G.L., Han, J.L., Liu, D.L., Sun, D.Y., et al. (2008). The calmodulin-binding protein kinase 3 is part of heat-shock signal transduction in *Arabidopsis thaliana*. *Plant J.* 55**,** 760-773. doi: 10.1111/j.1365-313X.2008.03544.x

Liu, H.T., Li, G.L., Chang, H., Sun, D.Y., Zhou, R.G., and Li, B. (2007). Calmodulin-binding protein phosphatase PP7 is involved in thermotolerance in *Arabidopsis*. *Plant Cell Environ.* 30**,** 156-164. doi: 10.1111/j.1365-3040.2006.01613.x

Liu, T.Y., Huang, T.K., Tseng, C.Y., Lai, Y.S., Lin, S.I., Lin, W.Y., et al. (2012). PHO2-dependent degradation of PHO1 modulates phosphate homeostasis in *Arabidopsis*. *Plant Cell* 24**,** 2168-2183. doi: 10.1105/tpc.112.096636

Liu, Z., Jia, Y., Ding, Y., Shi, Y., Li, Z., Guo, Y., et al. (2017). Plasma membrane CRPK1-mediated phosphorylation of 14-3-3 proteins induces their nuclear import to fine-tune CBF signaling during cold response. *Mol. Cell* 66**,** 117-128 e115. doi: 10.1016/j.molcel.2017.02.016

Lv, Q., Zhong, Y., Wang, Y., Wang, Z., Zhang, L., Shi, J., et al. (2014). SPX4 negatively regulates phosphate signaling and homeostasis through its interaction with PHR2 in rice. *Plant Cell* 26**,** 1586-1597. doi: 10.1105/tpc.114.123208

Ma, L., Ye, J., Yang, Y., Lin, H., Yue, L., Luo, J., et al. (2019). The SOS2-SCaBP8 complex generates and fine-tunes an AtANN4-dependent calcium signature under salt stress. *Dev. Cell* 48**,** 697-709.e695. doi: 10.1016/j.devcel.2019.02.010

Miura, K., Jin, J.B., Lee, J., Yoo, C.Y., Stirm, V., Miura, T., et al. (2007). SIZ1-mediated sumoylation of ICE1 controls *CBF3/DREB1A* expression and freezing tolerance in *Arabidopsis*. *Plant Cell* 19**,** 1403-1414. doi: 10.1105/tpc.106.048397

Navarro, C., Mateo-Elizalde, C., Mohan, T.C., Sánchez-Bermejo, E., Urrutia, O., Fernández-Muñiz, M.N., et al. (2021). Arsenite provides a selective signal that coordinates arsenate uptake and detoxification through the regulation of PHR1 stability in *Arabidopsis*. *Mol. Plant* 14**,** 1489-1507. doi: 10.1016/j.molp.2021.05.020

Olate, E., Jimenez-Gomez, J.M., Holuigue, L., and Salinas, J. (2018). NPR1 mediates a novel regulatory pathway in cold acclimation by interacting with HSFA1 factors. *Nat. Plants* 4**,** 811-823. doi: 10.1038/s41477-018-0254-2

Puga, M.I., Mateos, I., Charukesi, R., Wang, Z., Franco-Zorrilla, J.M., de Lorenzo, L., et al. (2014). SPX1 is a phosphate-dependent inhibitor of phosphate starvation response 1 in *Arabidopsis*. *Proc. Natl. Acad. Sci. U. S. A.* 111**,** 14947-14952. doi: 10.1073/pnas.1404654111

Ruan, W., Guo, M., Wang, X., Guo, Z., Xu, Z., Xu, L., et al. (2019). Two RING-finger ubiquitin E3 ligases regulate the degradation of SPX4, an internal phosphate sensor, for phosphate homeostasis and signaling in rice. *Mol. Plant* 12**,** 1060-1074. doi: 10.1016/j.molp.2019.04.003

Sato, H., Mizoi, J., Tanaka, H., Maruyama, K., Qin, F., Osakabe, Y., et al. (2014). *Arabidopsis* DPB3-1, a DREB2A interactor, specifically enhances heat stress-induced gene expression by forming a heat stress-specific transcriptional complex with NF-Y subunits. *Plant Cell* 26**,** 4954-4973. doi: 10.1105/tpc.114.132928

Sharma, A., Sharma, B., Hayes, S., Kerner, K., Hoecker, U., Jenkins, G.I., et al. (2019). UVR8 disrupts stabilisation of PIF5 by COP1 to inhibit plant stem elongation in sunlight. *Nat. Commun.* 10**,** 4417. doi: 10.1038/s41467-019-12369-1

Shi, J., Kim, K.N., Ritz, O., Albrecht, V., Gupta, R., Harter, K., et al. (1999). Novel protein kinases associated with calcineurin B-like calcium sensors in *Arabidopsis*. *Plant Cell* 11**,** 2393-2405. doi: 10.1105/tpc.11.12.2393

Straub, T., Ludewig, U., and Neuhauser, B. (2017). The kinase CIPK23 inhibits ammonium transport in *Arabidopsis thaliana*. *Plant Cell* 29**,** 409-422. doi: 10.1105/tpc.16.00806

Takahashi, Y., Zhang, J., Hsu, P.K., Ceciliato, P.H.O., Zhang, L., Dubeaux, G., et al. (2020). MAP3Kinase-dependent SnRK2-kinase activation is required for abscisic acid signal transduction and rapid osmotic stress response. *Nat. Commun.* 11**,** 12. doi: 10.1038/s41467-019-13875-y

Teige, M., Scheikl, E., Eulgem, T., Doczi, R., Ichimura, K., Shinozaki, K., et al. (2004). The MKK2 pathway mediates cold and salt stress signaling in *Arabidopsis*. *Mol. Cell* 15**,** 141-152. doi: 10.1016/j.molcel.2004.06.023

Tischer, S.V., Wunschel, C., Papacek, M., Kleigrewe, K., Hofmann, T., Christmann, A., et al. (2017). Combinatorial interaction network of abscisic acid receptors and coreceptors from *Arabidopsis thaliana*. *Proc. Natl. Acad. Sci. U. S. A.* 114**,** 10280-10285. doi: 10.1073/pnas.1706593114

Wang, X., Ding, Y., Li, Z., Shi, Y., Wang, J., Hua, J., et al. (2019). PUB25 and PUB26 promote plant freezing tolerance by degrading the cold signaling negative regulator MYB15. *Dev. Cell* 51**,** 222-235 e225. doi: 10.1016/j.devcel.2019.08.008

Wang, X.P., Chen, L.M., Liu, W.X., Shen, L.K., Wang, F.L., Zhou, Y., et al. (2016). AtKC1 and CIPK23 synergistically modulate AKT1-mediated low-potassium stress responses in *Arabidopsis*. *Plant Physiol.* 170**,** 2264-2277. doi: 10.1104/pp.15.01493

Xu, J., Li, H.D., Chen, L.Q., Wang, Y., Liu, L.L., He, L., et al. (2006). A protein kinase, interacting with two calcineurin B-like proteins, regulates K^+^ transporter AKT1 in *Arabidopsis*. *Cell* 125**,** 1347-1360. doi: 10.1016/j.cell.2006.06.011

Yamada, K., Fukao, Y., Hayashi, M., Fukazawa, M., Suzuki, I., and Nishimura, M. (2007). Cytosolic HSP90 regulates the heat shock response that is responsible for heat acclimation in *Arabidopsis thaliana*. *J. Biol. Chem.* 282**,** 37794-37804. doi: 10.1074/jbc.M707168200

Yan, M., Fan, X., Feng, H., Miller, A.J., Shen, Q., and Xu, G. (2011). Rice OsNAR2.1 interacts with OsNRT2.1, OsNRT2.2 and OsNRT2.3a nitrate transporters to provide uptake over high and low concentration ranges. *Plant Cell Environ.* 34**,** 1360-1372. doi: 10.1111/j.1365-3040.2011.02335.x

Yang, T., Shad Ali, G., Yang, L., Du, L., Reddy, A.S., and Poovaiah, B.W. (2010). Calcium/calmodulin-regulated receptor-like kinase CRLK1 interacts with MEKK1 in plants. *Plant Signal. Behav.* 5**,** 991-994. doi: 10.4161/psb.5.8.12225

Yang, Y., Liang, T., Zhang, L., Shao, K., Gu, X., Shang, R., et al. (2018). UVR8 interacts with WRKY36 to regulate HY5 transcription and hypocotyl elongation in *Arabidopsis*. *Nat. Plants* 4**,** 98-107. doi: 10.1038/s41477-017-0099-0

Ye, K., Li, H., Ding, Y., Shi, Y., Song, C., Gong, Z., et al. (2019). BRASSINOSTEROID-INSENSITIVE2 negatively regulates the stability of transcription factor ICE1 in response to cold stress in *Arabidopsis*. *Plant Cell* 31**,** 2682-2696. doi: 10.1105/tpc.19.00058

Ye, Q., Wang, H., Su, T., Wu, W.H., and Chen, Y.F. (2018). The ubiquitin E3 ligase PRU1 regulates WRKY6 degradation to modulate phosphate homeostasis in response to low-Pi stress in *Arabidopsis*. *Plant Cell* 30**,** 1062-1076. doi: 10.1105/tpc.17.00845

Yoshinari, A., Hosokawa, T., Amano, T., Beier, M.P., Kunieda, T., Shimada, T., et al. (2019). Polar localization of the borate exporter BOR1 requires AP2-dependent endocytosis. *Plant Physiol.* 179**,** 1569-1580. doi: 10.1104/pp.18.01017

Yuan, Y., Wu, H., Wang, N., Li, J., Zhao, W., Du, J., et al. (2008). FIT interacts with AtbHLH38 and AtbHLH39 in regulating iron uptake gene expression for iron homeostasis in *Arabidopsis*. *Cell Res.* 18**,** 385-397. doi: 10.1038/cr.2008.26

Yue, W., Ying, Y., Wang, C., Zhao, Y., Dong, C., Whelan, J., et al. (2017). OsNLA1, a RING-type ubiquitin ligase, maintains phosphate homeostasis in *Oryza sativa* via degradation of phosphate transporters. *Plant J.* 90**,** 1040-1051. doi: 10.1111/tpj.13516

Zhang, J., Gu, M., Liang, R., Shi, X., Chen, L., Hu, X., et al. (2021). OsWRKY21 and OsWRKY108 function redundantly to promote phosphate accumulation through maintaining the constitutive expression of *OsPHT1*;1 under phosphate-replete conditions. *New Phytol.* 229**,** 1598-1614. doi: 10.1111/nph.16931

Zhang, P., Wang, R., Ju, Q., Li, W., Tran, L.P., and Xu, J. (2019). The R2R3-MYB transcription factor MYB49 regulates cadmium accumulation. *Plant Physiol.* 180**,** 529-542. doi: 10.1104/pp.18.01380

Zhao, C., Wang, P., Si, T., Hsu, C.C., Wang, L., Zayed, O., et al. (2017). MAP kinase cascades regulate the cold response by modulating ICE1 protein stability. *Dev. Cell* 43**,** 618-629 e615. doi: 10.1016/j.devcel.2017.09.024

Zhao, C., Zayed, O., Yu, Z., Jiang, W., Zhu, P., Hsu, C.C., et al. (2018). Leucine-rich repeat extensin proteins regulate plant salt tolerance in *Arabidopsis*. *Proc. Natl. Acad. Sci. U. S. A.* 115**,** 13123-13128. doi: 10.1073/pnas.1816991115

Zheng, P., Cao, L., Zhang, C., Pan, W., Wang, W., Yu, X., et al. (2022). MYB43 as a novel substrate for CRL4(PRL1) E3 ligases negatively regulates cadmium tolerance through transcriptional inhibition of HMAs in *Arabidopsis*. *New Phytol.* 234**,** 884-901. doi: 10.1111/nph.18020

Zhong, Y., Wang, Y., Guo, J., Zhu, X., Shi, J., He, Q., et al. (2018). Rice SPX6 negatively regulates the phosphate starvation response through suppression of the transcription factor PHR2. *New Phytol.* 219**,** 135-148. doi: 10.1111/nph.15155
